# Aerobic Exercise Training Reduces Atherogenesis Induced by Low-Sodium Diet in LDL Receptor Knockout Mice

**DOI:** 10.3390/antiox11102023

**Published:** 2022-10-13

**Authors:** Ana Paula Garcia Bochi, Guilherme da Silva Ferreira, Vanessa Del Bianco, Paula Ramos Pinto, Letícia Gomes Rodrigues, Mayara da Silva Trevisani, Luzia Naoko Shinohara Furukawa, Kely Cristina Soares Bispo, Alexandre Alves da Silva, Ana Paula Pereira Velosa, Edna Regina Nakandakare, Ubiratan Fabres Machado, Walcy Paganelli Rosolia Teodoro, Marisa Passarelli, Sergio Catanozi

**Affiliations:** 1 Laboratorio de Lipides (LIM-10), Hospital das Clinicas (HCFMUSP) da Faculdade de Medicina da Universidade de São Paulo, São Paulo 01246 000, Brazil; 2 Laboratory of Renal Pathophysiology, Department of Internal Medicine, School of Medicine, University of São Paulo, São Paulo 01246 000, Brazil; 3 Department of Pathology, University of São Paulo Medical School, São Paulo 01246 000, Brazil; 4 Department of Physiology and Biophysics, Mississippi Center for Obesity Research, Cardiorenal and Metabolic Diseases Research Center, University of Mississippi Medical Center, Jackson, MS 39216, USA; 5 Rheumatology Division of the Hospital das Clinicas, University of São Paulo Medical School, São Paulo 01246 000, Brazil; 6 Department of Physiology and Biophysics, Institute of Biomedical Sciences, University of São Paulo, São Paulo 05508 000, Brazil; 7 Programa de Pós-Graduação em Medicina, Universidade Nove de Julho (UNINOVE), São Paulo 01525 000, Brazil

**Keywords:** low-sodium diet, atherogenesis, dyslipidemia, aerobic-exercise training, insulin resistance

## Abstract

This study investigated the efficacy of aerobic exercise training (AET) in the prevention of dyslipidemia, insulin resistance (IR), and atherogenesis induced by severe low-sodium (LS) diet. LDL receptor knockout (LDLR KO) mice were fed a low-sodium (LS) (0.15% NaCl) or normal-sodium (NS; 1.27% NaCl) diet, submitted to AET in a treadmill, 5 times/week, 60 min/day, 15 m/min, for 90 days, or kept sedentary. Blood pressure (BP), plasma total cholesterol (TC) and triglyceride (TG) concentrations, lipoprotein profile, and insulin sensitivity were evaluated at the end of the AET protocol. Lipid infiltration, angiotensin II type 1 receptor (AT1), receptor for advanced glycation end products (RAGE), carboxymethyllysine (CML), and 4-hydroxynonenal (4-HNE) contents as well as gene expression were determined in the brachiocephalic trunk. BP and TC and gene expression were similar among groups. Compared to the NS diet, the LS diet increased vascular lipid infiltration, CML, RAGE, 4-HNE, plasma TG, LDL-cholesterol, and VLDL-TG. Conversely, the LS diet reduced vascular AT1 receptor, insulin sensitivity, HDL-cholesterol, and HDL-TG. AET prevented arterial lipid infiltration; increases in CML, RAGE, and 4-HNE contents; and reduced AT1 levels and improved LS-induced peripheral IR. The current study showed that AET counteracted the deleterious effects of chronic LS diet in an atherogenesis-prone model by ameliorating peripheral IR, lipid infiltration, CML, RAGE, 4-HNE, and AT1 receptor in the intima-media of the brachiocephalic trunk. These events occurred independently of the amelioration of plasma-lipid profile, which was negatively affected by the severe dietary-sodium restriction.

## 1. Introduction

Reductions in sodium intake are recommended as important non-pharmacological therapy to help reduce blood pressure (BP) and cardiovascular diseases [1,2]. However, long-term severe low-sodium (LS) diet may cause undesirable effects including dyslipidemia, insulin resistance (IR), increased activity of the sympathetic nervous system (SNS), and activation of the renin-angiotensin-aldosterone system (RAAS), that may act in concert to favor atherogenesis [3].

Low-density lipoprotein receptor knockout (LDLR KO) mice, frequently used in atherosclerosis investigations, are deficient in LDL receptor which impairs the rate of very low-density lipoprotein (VLDL) and LDL removal from the plasma; thus, favoring hyperlipidemia and moderate vascular lesions (atherogenesis) even when fed a normal low-fat rodent chow [4]. Normotensive LDLR KO or apolipoprotein E knockout (apoE KO) mice chronically fed a LS diet showed enhanced hyperlipidemia and arterial-wall lipid infiltration [5,6,7]. In addition, LS diet intensified the hyperlipidemia and prompted greater arterial wall glycoxidation and lipid infiltration in simultaneously hypertensive and hyperlipidemic mice [8]. These mice also showed increased arterial amounts of the advanced glycation end-product, carboxymethyllysine (CML), and its receptor, RAGE, which occurred independently of the presence of established diabetes mellitus (DM) [8].

Aerobic exercise training (AET) has been demonstrated to improve lipid profile, and to ameliorate reverse cholesterol transport, insulin sensitivity, and antioxidant defenses in the arterial wall, contributing to reduced atherogenesis and to increased life span [9,10,11,12,13]. Thus, we hypothesized that these beneficial effects of AET may mitigate the potential adverse effects of LS diet in situations where there is increased risk for development of atherosclerosis. In the current study, we investigated whether AET performed for 90 days prevents or at least significantly attenuates the development of hyperlipidemia and atherogenesis in LDLR KO mice fed LS chow. We found that chronic LS diet worsened hypertriglyceridemia, IR, and pro-atherogenic lipoprotein (LP) profile as well as glycoxidation, lipid infiltration, and peroxidation in the brachiocephalic trunk of LDLR KO mice, and that AET was very efficacious in preventing these effects.

## 2. Materials and Methods

### 2.1. Animals

Homozygous LDLR KO mice, inbred on a C57BL/6J, were purchased from the Jackson Laboratory (Bar Harbor, ME, USA), and a breeding colony was established in-house. The experimental protocol was approved by the Animal Care and Research Advisory Committee of the Faculdade de Medicina da Universidade de Sao Paulo (CEUA # 149/16) and was strictly performed according to the U.S. National Institutes of Health Guide for the Care and Use of Laboratory Animals. Animals were housed in a conventional animal facility at 22 ± 2 °C under a 12 h light/dark cycle with free access to pelleted commercial chow (Nuvilab CR1—Nuvital Nutrients, Colombo, PR, Brazil) and drinking water. Blood samples (200 µL) were drawn from the tail vein into heparinized micro-hematocrit capillary tubes after a 12 h fasting period. Mice were euthanized by single intraperitoneal overdose injection of sodium thiopental (150 mg/kg of body mass: Thiopentax^®^).

### 2.2. Experimental Protocol

Twelve-week-old male LDLR KO mice were fed ad libitum pelleted chow containing the following nutrients (g/100 g): casein (28.7); sucrose (31.3); cornstarch (20.0); soybean oil (6.0); minerals and vitamins. Animals were fed either a low-sodium (LS; 0.06% sodium = 0.15% NaCl; Envigo Teklad Diets— Indianapolis, IN, USA—TD 92141) diet or a normal-sodium (NS; 0.5% sodium = 1.27% NaCl; Envigo Teklad Diets—Indianapolis, IN, USA—TD92140) diet. Cellulose replaced amounts of sodium in the diet. After homogenizing the groups of animals according to their baseline parameters (maximum exercise capacity, body mass (BM), BP, total cholesterol (TC), and triglycerides (TG)), mice were assigned into four experimental groups (*n* = 20 animals/group) according to different concentrations of sodium in the diets and AET or sedentarism: (1) fed NS diet and kept sedentary (NS-S); (2) trained (NS-T); (3) fed LS diet and kept sedentary (LS-S); or (4) trained (LS-T).

The LS diet contained the minimum amount of sodium required for normal development in rodents. Food intake per group and BM of each animal were monitored weekly from the beginning until the end of the protocol (90-day experimental protocol). Plasma TC and TG concentrations, hematocrit, BM, systolic blood pressure (SBP), heart rate (HR), and maximum exercise capacity were carried out before and at the end of the 90-day protocol. Insulin tolerance test (ITT), 24 h urinary sodium excretion (UNa), and LP profile were determined at the end of the experimental protocol. After euthanasia, the brachiocephalic trunk was resected for histological and gene-expression analyses.

See the Supplementary Materials for further information.

### 2.3. Aerobic Exercise Training

One week before the beginning of the AET protocol, the animals were acclimated to the treadmill by exposing them to a speed of 12 m/min and a gradual increase in time (10 min) per day from 30 to 60 min. The AET was performed on a specially designed treadmill for mice (WEG, São Carlos, SP, Brazil), five times per week, at 15 m/min, 60 min per day, for 90 days [14]. There was exclusion of animals that had not acclimated to running on the treadmill (~10%). The exercise intensity was determined as 60% of the maximum exercise capacity (moderate exercise intensity), as previously reported [15]. The maximum exercise capacity test was performed at baseline and at the end of the 90-day protocol, as previously described [15]. Briefly, the maximum exercise capacity test consisted of an initial speed at 9 m/min, 0% inclination, and increases of 3 m/min every 3 min until complete inability to run. The maximum running time of each animal was used as the parameter of physical conditioning.

### 2.4. Insulin Tolerance Test

At the end of the experimental protocol, the mice were fasted for 4 h and received a single intraperitoneal injection of insulin (1 U/kg of BM; Humulin R, Eli Lilly—Sao Paulo-Brazil). Blood samples were drawn from the tail vein for determination of blood glucose using a glucometer (Accu Check Performa—Roche, Brazil) every 10 min, for 30 min. The blood glucose decay rate (kITT) was determined by linear regression between baseline and 30 min [16].

### 2.5. Brachiocephalic Trunk Isolation and Histomorphometry

After euthanasia, the heart and vascular tree were dissected with the use of a dissecting microscope and transcardially perfused under low-pressure, with cold 0.9% NaCl solution to clear the lumen of blood. For histological analyses, tissue samples were also perfused with tissue-freezing medium (Tissue-Tek-OCT Compound, USA). The brachiocephalic trunk was excised in the fresh state along with a patch of the aortic arch and a segment of the right subclavian and right common carotid arteries for appropriate orientation during histological processing. Then, the brachiocephalic trunk was wrapped around with tissue-freezing medium and immediately frozen in liquid nitrogen. Serial 2-μm-thick cross-sections were attained by using a cryostat (Leica, model CM1800—Nussloch, Germany ) [17]. Histological sections were assigned either for lipid infiltration analysis or immunofluorescence assays. Lipid infiltration, CML—the main advanced glycation end product (AGE), receptor for advanced glycation end products (RAGE), 4-hydroxynonenal (4-HNE)—a lipid peroxidation marker—and angiotensin II (ANG II) type-1(AT1) receptor contents were assessed in the cross-sectional areas of brachiocephalic trunk comprising the intima and media layers. Digital images of either positively stained or immunostained sections from the intima to the external elastic lamina were blindly evaluated using an optical microscope (Olympus-BX51, Tokyo, Japan), connected to a video camera (Olympus Co, St Laurent, QC, Canada) and to an image-analysis software (Image-Pro Plus 6.0). Four to five histological fields of each artery were delimited in each field using 400× magnification. Data are expressed as the mean percentage of the total positively stained or immunostained area of the brachiocephalic trunk cross-sections [18].

### 2.6. Oil Red O Staining

Lipid infiltration in the arterial wall was assessed by Oil Red O staining of cross-sections (each 2 μm thick), as previously described [18].

### 2.7. Immunofluorescence Staining

Histological sections were incubated for 48 h, at 4 °C, with rabbit polyclonal antibodies anti-CML (1:30; Cat # ab27684), anti-AT1 receptor (1:50; Cat # ab18801), anti-4-HNE (1:50; Cat # ab46545) (Abcam, Cambridge, UK) and anti-RAGE (1:10; Cat # 600-401-P67) (Rockland Immunochemical Inc, Limerick, PA, USA) diluted in 1% bovine serum albumin (BSA) and 0.5% Triton. Samples were washed in phosphate-buffered saline (PBS) and 0.005% Tween 20 and incubated for 60 min at room temperature with Alexa Fluor^®^ 488-conjugated goat anti-rabbit IgG antibody (Invitrogen, Fisher Scientific Baltics UAB, Cat # A11008, Vilnius, Lithuania) diluted at 1:200 in a PBS solution containing 0.003% Evans blue dye. After washing with PBS and Tween 20, the sections were incubated for 15 min at room temperature with 4,6-diamidino-2-phenylindole, dihydrochloride (DAPI) diluted at 1:200 in PBS.

See the Supplementary Materials for further information.

### 2.8. Real-Time Quantitative Polymerase Chain Reaction (RT-qPCR) in Brachiocephalic Trunk

For gene expression analyses, the brachiocephalic trunk was stored in RNAlater™ Stabilization Solution (Thermofisher Scientific, Waltham, MA, USA) at 4 °C, for 24 h, and then at −80 °C. Briefly, samples were mechanically homogenized using a Tissue-Tearor homogenizer model 985370 (Biospec, OK, USA), using a frozen cup with liquid nitrogen, rotating at 1500 rpm for 1 min, resuspended in 1000 µL of TRIzol Invitrogen (Life Technologies Corporation, Carlsbad, CA, USA), and added to 200 µL of chloroform (Merck, São Paulo, Brazil). After centrifugation at 13,000 g for 15 min, the aqueous upper phase was carefully removed and transferred to the extraction column of the Isolation Qiagen RNeasy mini columns RNA isolation kit (Qiagen GmbH, Hilden, Germany) and the protocol followed according to the manufacturer’s guidelines. The presence of bands corresponding to 18 s and 28 s ribosomal RNA and total RNA integrity and quality were assessed by 2100 Bioanalyzer (Agilent Technologies, Santa Clara, CA, USA), using Agilent RNA 6000 Pico kit (Agilent Technologies, Waldbronn, Germany). cDNA was obtained from 1000 ng of total RNA using a commercial high-capacity RNA-to-cDNA kit (Applied Biosystems, Thermofisher Scientific, Waltham, MA, USA). The volume of cDNA was then diluted 10x in endonuclease-free water and stored at −20 °C. mRNA expression was measured by real-time quantitative polymerase chain reaction (RT-qPCR) using TaqMan probes (Applied Biosystems, Thermofisher Scientific, Waltham, MA, USA) in Step One Plus Real-Time qPCR System (Applied Biosystems, Thermofisher Scientific, Waltham, MA, USA): *Agtr1a*, angiotensin II type 1a receptor (Mm00616371_m1); *Ager*, receptor for advanced glycation end products (Mm00545815_m1); *Orl1*, lectin-like oxidized low-density lipoprotein receptor-1 (Mm00454588_m1). According to the geNorm (http://leonxie.com/referencegene.php, Accessed on 2 February 2021) program, glyceraldehyde-3-phosphate dehydrogenase—*Gapdh—*(Mm99999915_m1) was selected as the housekeeping gene stable for arterial tissue analyses [19].

### 2.9. Statistical Analyses

Statistical analyses were performed using Minitab 19 Software (Minitab LLC, State College, PA, USA). Kruskal–Wallis and one-way ANOVA tests were used, respectively, to assess data normality and compare the baseline findings among groups. The two-way ANOVA test (generalized linear model) with two fixed factors (diet and exercise) and Tukey’s post-test were applied for comparisons among data attained after experimental interventions. The Kolmogorov–Smirnov test was used to assess data normality. Statistical differences express either diet (NS vs. LS) or AET effect (S vs. T) or interaction of both factors. *p*-values < 0.05 were considered statistically significant. Data are expressed as mean ± standard deviation (SD).

## 3. Results

### 3.1. Impact of Dietary Sodium Restriction and Aerobic Exercise Training Effects on Plasma Lipids, Hematocrit, and Body Mass of LDLR KO Mice

At baseline, plasma TC and TG levels, hematocrit, BM, SBP, and HR were similar among groups (Table 1).

As expected, at the end of the 90-day experimental protocol, mice fed the LS diet showed lower UNa when compared to NS groups, and mice that underwent AET showed increased maximum exercise capacity compared to sedentary mice, proving the effectiveness of both interventions (Table 1). Long-term dietary sodium restriction elicited higher plasma TG concentration as compared to the NS groups (Table 1).

**Figure 1:**
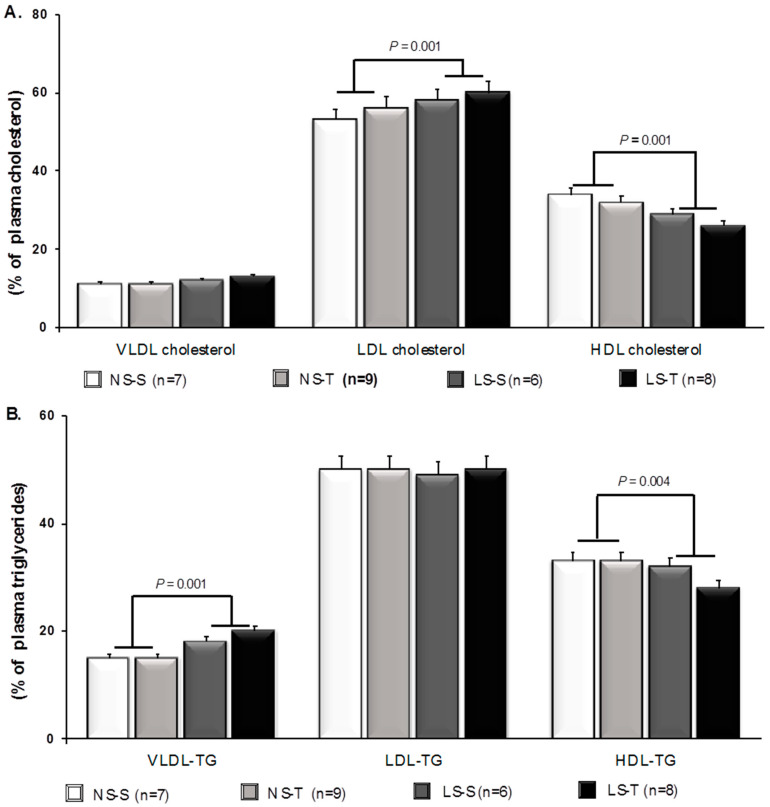
Percent of plasma lipids in lipoproteins by fast protein liquid chromatography (FPLC) in LDLR KO mice fed either a normal-sodium (NS) or a low-sodium (LS) diet, trained (T) or sedentary (S), after 90 days. (**A**) Percent of cholesterol in lipoprotein fractions. (**B**) Percent of triglycerides (TG) in lipoprotein fractions. Two-way ANOVA test and Tukey’s post-test were applied for comparisons among data. Results are expressed as mean ± standard deviation. *p* values show differences between diets. n = number of mice.

The fast protein liquid chromatography (FPLC) analysis showed that, as compared to the NS diet, the LS chow changed the plasma LP profile bringing about cholesterol accumulation in the LDL and reduction in the high-density lipoprotein (HDL) (Figure 1A). In addition, sodium restriction enhanced TG content in the VLDL and decreased in the HDL forms (Figure 1B). AET did not attenuate the adverse plasma lipid changes prompted by LS intake (Figure 1A,B).

BM was higher in LS-T mice as compared to other groups (Table 1); TC, hematocrit, SBP, and HR were similar among groups (Table 1). Plasma lipid profile, hematocrit, BM, SBP, and HR data were recently published [20,21].

### 3.2. Aerobic Exercise Training Prevented Dietary Sodium Restriction-Induced Insulin Resistance

As previously reported, the ITT evidenced an impaired blood glucose decay rate in the LS-S mice [20] when compared to the other groups and such adverse effect was prevented by the AET [21] (Table 1).

### 3.3. Aerobic Exercise Training Prevented Dietary Sodium Restriction-Induced Arterial Injury

**Figure 2:**
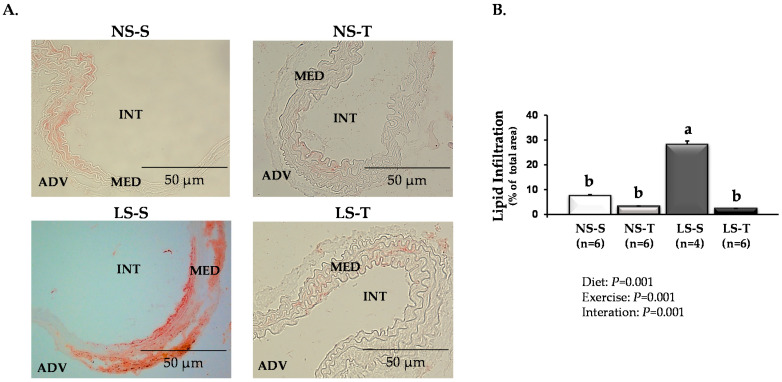
Vascular lipid infiltration in LDLR KO mice fed either a normal-sodium (NS) or a low-sodium (LS) diet, trained (T) or sedentary (S), after 90 days. (**A**) Representative micrographs of the brachiocephalic trunk stained with Oil Red O. The red staining indicates lipid infiltration. ADV, adventitia; MED, media; INT, intima (400×). (**B**) Histomorphometric analysis of Oil Red O-stained lipid infiltration in the brachiocephalic trunk. Two-way ANOVA test with two fixed factors (diet and exercise) and Tukey’s post-test were applied for comparisons among data. Results are expressed as mean ± standard deviation. Diet: *p* = 0.001; exercise: *p* = 0.001; interaction: *p* = 0.001. Distinct letters represent differences among groups (*p* < 0.05). *n* = number of mice.

The AET effects on long-term dietary sodium restriction-induced vascular injury were investigated in the LDLR KO mice. The Oil Red O staining revealed that AET inhibited the increased lipid infiltration induced by LS diet into the intima and media layers of the brachiocephalic trunk (Figure 2).

**Figure 3:**
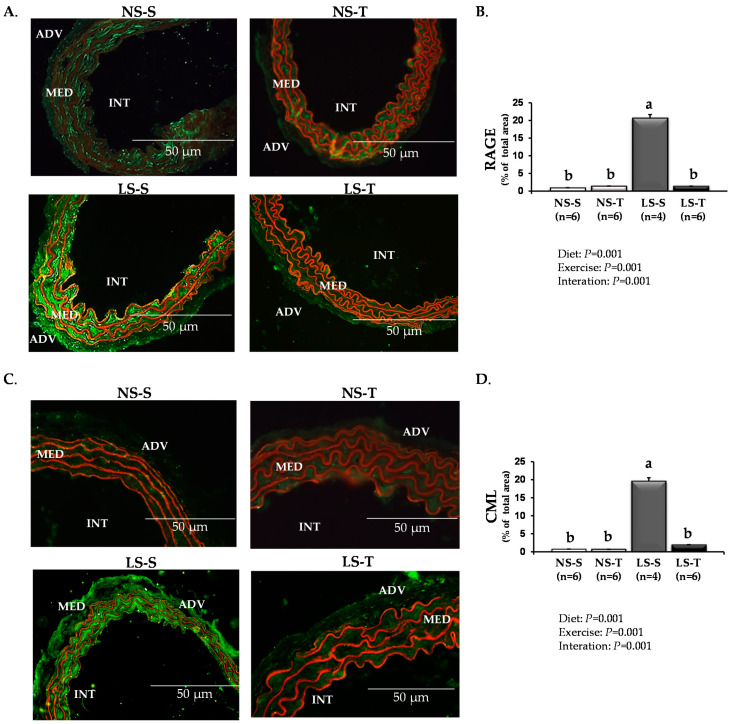
Vascular receptor for advanced glycation end products (RAGE) and carboxymethyllysine (CML) in LDLR KO mice fed either a normal-sodium (NS) or a low-sodium (LS) diet, trained (T) or sedentary (S), after 90 days. (**A**) Representative micrographs of immunofluorescence-stained RAGE in the brachiocephalic trunk. The green immunostaining indicates the RAGE expression. (**B**) Histomorphometric analysis of immunofluorescence-stained RAGE. (**C**) Representative micrographs of immunofluorescence-stained CML in the brachiocephalic trunk. The green immunostaining indicates the CML expression. (**D**) Histomorphometric analysis of immunofluorescence-stained CML. ADV, adventitia; MED, media; INT, intima (400×). Red staining indicates counterstaining by 0.006% Evans blue dye. Two-way ANOVA test with two fixed factors (diet and exercise) and Tukey’s post-test were applied for comparisons among data. Results are expressed as mean ± standard deviation. Distinct letters represent differences among groups (*p* < 0.05). *n* = number of mice.

**Figure 4:**
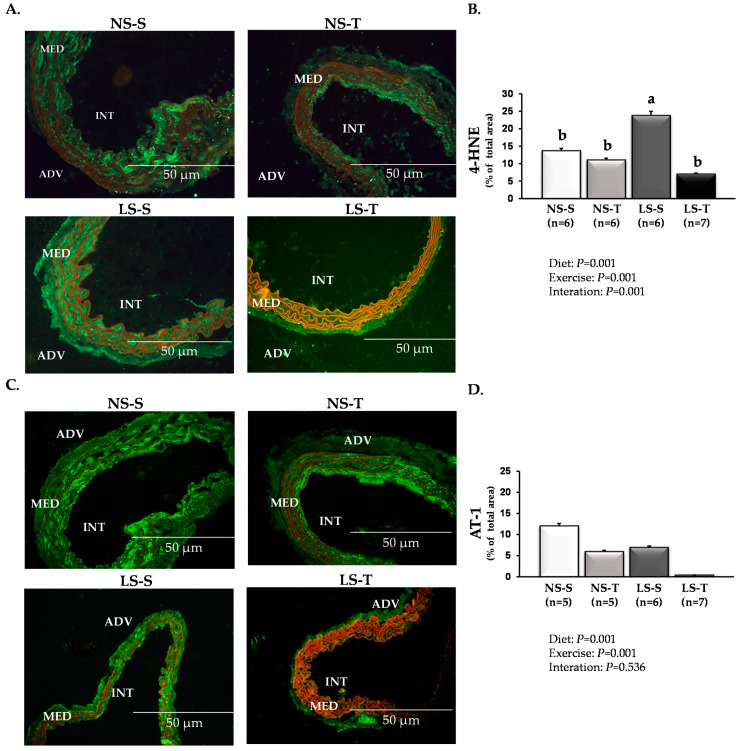
Vascular 4-hydroxynonenal (4-HNE) and angiotensin II type 1 receptor (AT1) receptor in LDLR KO mice fed either a normal-sodium (NS) or a low-sodium (LS) diet, trained (T) or sedentary (S), after 90 days. (**A**) Representative micrographs of immunofluorescence-stained 4-HNE in the brachiocephalic trunk. The green immunostaining indicates 4-HNE expression. (**B**) Histomorphometric analysis of immunofluorescence-stained 4-HNE. (**C**) Representative micrographs of immunofluorescence-stained AT1 receptor in the brachiocephalic trunk. The green immunostaining indicates AT1 receptor expression. (**D**) Histomorphometric analysis of immunofluorescence-stained AT1 receptor. ADV, adventitia; MED, media; INT, intima (400×). Red staining indicates counterstaining by 0.006% Evans blue dye. Two-way ANOVA test with two fixed factors (diet and exercise) and Tukey’s post-test were applied for comparisons among data. Results are expressed as mean ± standard deviation. Distinct letters represent differences among groups (*p* < 0.05). *n* = number of mice.

Similarly, immunofluorescence assays showed that AET prevented the enhancement of RAGE (Figure 3A,B), CML (Figure 3C,D), and 4-HNE (Figure 4A,B) in the arterial wall caused by the LS diet.

Accordingly, in addition to circumventing the local premature lipid infiltration, AET prevented arterial accumulation of lipid peroxidation marker and CML induced by the LS diet. AT1-positive staining was lower in arterial walls of LS-S and LS-T mice when compared to NS-S and NS-T, respectively, (Figure 4C,D).

The analysis also showed lower AT1 receptor content in LS-T and NS-T mice when compared to LS-S and NS-S groups, respectively, (Figure 4C,D).

### 3.4. Gene Expression

The expression of *Agtr1a*, *Ager*, and *Orl1* genes was not different among groups (Table 2).

## 4. Discussion

The present study demonstrated that AET counteracts the effects of chronically severe LS intake to induce hyperlipidemia and lipid infiltration in the brachiocephalic trunk of LDLR KO mice. Our results also suggest that AET reduced oxidative stress and advanced glycation in the arterial wall compartment, and improved peripheral insulin sensitivity, which together may have contributed to the attenuation of lipid infiltration in LS-T mice when compared to their sedentary counterparts. Consistent with previous reports from our group [5,8,22], chronic dietary sodium restriction induced IR, higher BM (in the LS-T group) and plasma TG levels. In this regard, the ITT revealed an impaired blood-glucose decay rate in the LS-S group, reflecting decreased insulin sensitivity [23,24]. Furthermore, as shown by FPLC analysis, there was increased LDL-C and VLDL-TG with a concomitant reduction in the HDL-C and HDL-TG, evidencing a pro-atherogenic plasma lipid profile. Although there was no difference in the cholesterolemia, the pro-atherogenic LP profile dramatically favored the early lipid accumulation in the arterial wall of the LS-S mice. TC, hematocrit, and SBP were not different among groups. Thus, these factors did not appear to influence the adverse plasma lipid profile or vascular damage elicited by LS intake.

The AT1 receptor signal transduction triggered by ANG II negatively modulates insulin signaling by stimulating the phosphorylation of serine residues in the beta subunit of the insulin receptor, insulin receptor substrate-1 (IRS-1), and phosphatidylinositol 3-kinase (PI3-kinase) [25]. Conversely, it has been suggested that ANG II-induced insulin resistance may involve enhanced oxidative stress, which inhibits insulin signaling downstream from PI3-kinase activation [26]. In the current study, AET prevented the insulin resistance induced by dietary sodium restriction. Substantial evidence supports the fact that exercise reduces inflammatory status, increases mitochondrial fatty acid oxidation, and improves insulin signaling [27,28].

The binding of ANG II to its AT1 receptor stimulates the activity of the enzymatic complex nicotinamide adenine dinucleotide phosphate (NADPH) oxidase and, consequently, increases reactive oxygen species (ROS) production [29]. ANG II-induced oxidative stress decreases AT1 receptor expression in vascular smooth muscle cells (VSMC) [30]. Furthermore, ANG II-mediated AT1 receptor internalization and desensitization and ANG II-induced long-term down-regulation of the AT1 receptor expression are mechanisms that reduce vascular AT1 receptor activity and density [31,32,33]. These findings corroborate the results of this study, in which AT1 receptor content was lower in the arterial wall of the LS mice than the NS groups. The mechanisms by which regular exercise reduces vascular AT1 receptor expression have not been fully elucidated. It has been shown that such effects are associated with decreased expression and activity of the enzymatic complex NADPH oxidase and reduction in vascular ROS biosynthesis [34]. Furthermore, in spontaneously hypertensive rats, exercise training increased the aortic levels of microRNA (miRNA)-155, which negatively modulates aortic mRNA levels and protein expression of AT1 receptor [35]. These findings show that part of the anti-atherogenic effects of physical exercise can be mediated by reduction of the AT1 receptor content, which leads to decreased vascular oxidative stress, lower expression of inflammatory molecules such as ICAM-1, VCAM-1, and MCP-1, and mitigation of local inflammatory activity, cell adhesion, and endothelial dysfunction [30,34]. Such findings support the results of the present study, considering that vascular AT1 receptor expression was lower in trained as compared to sedentary mice (LS-T vs. LS-S and NS-T vs. NS-S), denoting vasculoprotection, which was evidenced by reduced lipid infiltration and content of CML, RAGE, and 4-HNE in the arterial wall of LS-T mice. These mechanisms reveal that part of the vasculoprotective effects of AET may be mediated by reduction in vascular AT1 receptor content [36]. Accordingly, sedentary apoE/AT1 receptor-double-KO mice showed smaller atherosclerotic lesion size than apoE KO mice, independently of BP, TC, or HDL-C, demonstrating that AT1 receptor signaling contributes greatly to the atherogenesis [37]. In the present study, the lower atherogenesis evidenced in the LS-T mice may be attributed to the reduction of vascular AT1 receptor expression associated with other health benefits of regular exercise, such as anticoagulant, anti-inflammatory, and antioxidant effects that promote vascular protection [38,39,40,41,42,43,44,45,46,47,48]. AT1, RAGE, and lectin-like oxidized LDL receptor-1 (LOX-1; oxidized LDL receptor) gene expressions were not different among groups. In this sense, it has been reported that, although the concentrations of proteins correlate with the content of their respective mRNAs, frequently, such associations are not considered strong. Furthermore, gene transcription and translation usually do not show a linear and simple relationship [49,50].

The arterial wall of the LS-S mice revealed higher content of CML, RAGE, and 4-HNE when compared to the other experimental groups, which was prevented by AET. This evidences an important protective effect of AET against arterial injury induced by long-term severe sodium restriction. Thus, in the present study, the lower atherogenesis evidenced in the LS-T mice may be attributed to the reduced vascular levels of CML, RAGE, and 4-HNE. AT1 receptor signaling triggers oxidative stress, inflammatory response, and lipid peroxidation [51,52], which favor the CML generation and RAGE expression in the arterial wall in a positive-feedback loop, as previously demonstrated in sodium restriction [8]. It has been reported that the interplay between the RAAS signaling pathways and the AGE/RAGE axis sets up an effective positive-feedback loop that promotes arterial dysfunction and injury [53,54,55]. In this regard, chronic infusion of ANG II in Sprague–Dawley rats increased plasma and renal concentration of AGE, effects that were antagonized by pyridoxamine, an inhibitor of AGE generation [55]. Concurrently, continuous infusion of glycated rat serum albumin increased renal expression of angiotensinogen, angiotensin-converting enzyme, renin, and AT1 receptor, which were prevented by valsartan, a selective AT1 receptor antagonist [55]. Furthermore, LS diet did not increase atherosclerotic plaque area, markers of vascular inflammation and endothelial adhesion of leukocytes in the aorta of apoE/RAGE-double-KO mice as compared with apoE KO mice, demonstrating a crosstalk between AT1 receptor and RAGE in a model of ANG II-dependent atherogenesis [56]. In the same report, it was shown that ANG II binding to AT1 receptor elicited transactivation of RAGE and proinflammatory signaling events through a heteromeric AT1 receptor-RAGE complex, independently of RAGE ligands [56]. Although the aim of the present study was not to investigate the regulatory pathways previously described, the results regarding the vascular content of lipids, CML, RAGE, and 4-HNE suggest that severe dietary-sodium restriction induces the activity of such mechanisms in the murine arterial wall.

Damage in macrophage cholesterol homeostasis induced by advanced glycation has been demonstrated in animal models of DM and in cells cultured in hyperglycemic medium or with advanced glycated proteins [57,58,59]. It is interesting that independently of DM, chronically infused AGE-albumin triggers IR in healthy rats [60,61] and induces aortic lipid accumulation in non-diabetic dyslipidemic mice. The latter is prevented by inhibiting AT1 receptor with losartan to diminish oxidative stress, inflammation, and compounds of the AGE-RAGE axis [19]. It is worth noting that the atherogenic effects of LS diet are multiple, including its capacity to induce glycoxidative stress that impairs cholesterol efflux from arterial wall macrophages and its flux to the liver by reverse cholesterol transport.

In this study, we investigated the early stages of atherogenesis since differences in the sodium chloride concentrations in both experimental diets do not induce complex and advanced atherosclerotic lesions in LDLR KO mice. In the same animals, LS diet changed the glycerophospholipid and fatty acid profile in the gastrocnemius muscle, contributing to disorders of glycolipid homeostasis and peripheral insulin resistance [20]. Additionally, dietary sodium restriction favored TG accumulation in the liver and expression of genes related to synthesis, uptake, and metabolism of hepatic lipids [21].

Our findings showed that chronic dietary sodium restriction elicited IR and hypertriglyceridemia, and increased LDL-C and VLDL-TG with concurrent decreased HDL-C and HDL-TG. Additionally, LS diet enhanced lipid infiltration, CML, RAGE, and 4-HNE contents and lowered the AT1 receptor expression in the arterial wall. Despite AET not preventing the pro-atherogenic lipid profile induced by long-term severe sodium restriction, it improved insulin sensitivity and generated vasculoprotective responses. Although such evidence must be properly demonstrated in humans, the current study provided critical data that AET may be an attractive approach to inhibiting or mitigating the adverse effects promoted by severe low-sodium intake, which may enhance the beneficial actions of dietary-sodium restriction particularly in states of hypertension, heart failure, or kidney disease.

The present study also had its limitations. For instance, dietary sodium restriction promoted important adverse effects on systemic glycolipid metabolism and vascular injuries, many of which were inhibited by AET, however, we did not examine intracellular signaling pathways that modulate the demonstrated biological responses presented in our study. Previous studies showed a complex crosstalk between the RAAS and the AGE/RAGE axis [53,54,55,56], however, such signaling pathways were not explored and were just inferred in this experimental model as a theoretical basis to support the results from the present study.

Part of this referred limitation is associated with the small size of the murine brachiocephalic trunk (∼2 mm long; diameter of ∼0.5 mm; wall thickness of 0.04 mm) [62,63]. This vessel is particularly sensitive to atherogenesis with injuries that feature similar characteristics of clinical stages of lesions to those in humans [64], which facilitates histological analyses of early arterial wall lipid infiltration, as shown in the current study. However, its small size markedly limited the availability of samples to assess intracellular signaling pathways. Thus, further studies are necessary to investigate the intricate cell signaling that modulates the vascular biological responses to severe dietary sodium restriction.

The complexity of glycolipid metabolism and the pathophysiology of human dyslipidemia, insulin resistance, and metabolic syndrome make it extremely difficult to establish accurate animal models for investigations of atherosclerotic cardiovascular diseases [65]. Different murine models have been used to study the mechanisms of such diseases [65]. However, all of them offer advantages, disadvantages, or limitations according to the objectives of the study and, thus, it is challenging to determine which experimental model to use for investigating the dietary sodium restriction-induced systemic effects and their cardiovascular implications. The LDLR KO mouse model enables investigation of the adverse effects of dietary sodium restriction in the arterial wall under reduced influences of some important confounding factors. This is particularly important due to the fact that in wild-type mice, the use of a high-fat diet containing cholic acid to induce atherosclerosis can cause hepatotoxicity and inflammation. In future studies, we also plan to examine whether female mice exhibit a similar phenotype in response to both LS diet and AET to that observed in males, or whether there are sex differences.

## 5. Conclusions

In conclusion, our findings reveal that AET counteracts the deleterious effects of chronic LS diet in atherogenesis by reducing peripheral IR, lipid infiltration, CML, RAGE, 4-HNE, and AT1 receptor in the intima-media of the brachiocephalic trunk in LDLR KO mice. These events occurred independently of the amelioration in plasma lipid profile, which was negatively affected by severe dietary sodium restriction.

## Figures and Tables

**Table 1:** Body mass, biochemical plasma, hematocrit, heart rate, blood pressure, urinary sodium, and exercise capacity of LDLR KO mice fed either a normal-sodium (NS) or a low-sodium (LS) diet, trained (T) or sedentary (S), at baseline and after 90 days.

	NS				LS				*p*	
	Baseline NS-S	Sedentary NS-S	Baseline NS-T	AET NS-T	Baseline LS-S	Sedentary LS-S	Baseline LS-T	AET LS-T	Diet	AET
BM (g) (n = 15, 19, 15, 20)	26 ± 2	25 ± 2	25 ± 1	24 ± 2	25 ± 2	24 ± 1	25 ± 2	27 ± 2	0.006	0.02
TC (mmol/L) (n = 12, 14, 13, 13)	6.9 ± 0.9	8.8 ± 3.7	6.7 ± 0.9	8.6 ± 2.8	7.4 ± 1.0	8.6 ± 2.8	6.9 ± 1.0	9.6 ± 2.7	-	-
TG (mmol/L) (n = 12, 14, 13, 13)	1.9 ± 0.4	1.4 ± 0.3	1.8 ± 0.4	1.5 ± 0.6	1.8 ± 0.3	1.7 ± 0.4	1.8 ± 0.4	2.1 ± 0.7	0.013	-
Hematocrit (%) (n = 9, 7, 8, 9)	49 ± 5.4	48 ± 2	50 ± 7.0	49 ± 2	49 ± 6.3	50 ± 2	49 ± 6.2	49 ± 3	-	-
HR (bpm) (n = 8, 8, 8, 9)	515 ± 76	552 ± 63	487 ± 56	531 ± 50	502 ± 63	541 ± 66	481 ± 80	544 ± 65	-	-
SBP (mmHg) (n = 8, 8, 7, 9)	108 ± 8	108 ± 7	113 ± 6	105 ± 5	111 ± 6	101 ± 5	109 ± 6	103 ± 5	-	-
UNa (mEq/24 h) (n = 8, 7, 6, 6)		0.157 ± 0.021		0.186 ± 0.034		0.027 ± 0.011		0.030 ± 0.020	0.001	-
Glucose decay rate (%/min) (n = 7, 8, 6, 5)		3.46 ± 0.87		3.48 ± 0.76		1.89 ± 0.32		3.43 ± 1.01	0.015	0.018
Maximum exercise capacity (s) (n = 5, 6, 6, 7)		947 ± 252		1844 ± 317		1165 ± 177		1740 ± 531	-	0.001

AET, aerobic exercise training; BM, body mass; HR, heart rate; SBP, systolic blood pressure; TC, total cholesterol; TG, triglycerides; UNa, urinary sodium excretion. Results are expressed as mean ± standard deviation (SD). Two-way ANOVA with Tukey’s post-test. n = number of mice.

**Table 2:** The expression of *Agtr1a*, *Ager,* and *Orl1* genes in LDLR KO mice fed either a normal-sodium (NS) or a low-sodium (LS) diet, trained (T) or sedentary (S), after 90 days.

	NS		LS		*p*		
	Sedentary NS-S	AET NS-T	Sedentary LS-S	AET LS-T	Diet	AET	Interaction
*Agtr1a* (2^^−ΔΔCT^) (n = 5)	1.00	1.99	1.24	2.33	0.714	0.215	0.942
*Ager* (2^^−ΔΔCT^) (n = 5)	1.00	0.66	0.46	0.67	0.360	0.968	0.337
*Orl1* (2^^−ΔΔCT^) (n = 5)	1.00	0.55	0.44	0.31	0.223	0.926	0.257

Relative expression (2^^−ΔΔCT^) of the *Agtr1a* (encoding for angiotensin II receptor type 1), *Ager* (encoding for advanced glycation end product-specific receptor), and *Orl1* (encoding for lectin-like oxidized LDL receptor-1; oxidized low-density lipoprotein receptor) genes. Gene expression was evaluated in brachiocephalic trunk homogenates by RT-qPCR. Gapdh (glyceraldehyde-3-phosphate dehydrogenase) was used as a reaction-normalizing gene. Gene expression was calculated by the formula: 2-((CT of the target gene—mean endogenous control CT)—calibrator), and the mean CT of the NS-S group as calibrator (2^^−ΔΔCT^). Results presented as amplitude of expression and standard deviation were compared by two-factor ANOVA test with two fixed factors (diet and exercise) with Tukey’s post-test. *n* = number of mice.

## Data Availability

The raw data supporting the conclusions of this article will be made available by the authors, without undue reservation.

## Supplementary Materials

The following are available online at https://www.mdpi.com/article/10.3390/antiox11102023/s1. Table S1: Antibodies used in the immunofluorescence staining; Table S2: Description of the experimental diets.
