# Supplementary material for: Aerobic Exercise Training Reduces Atherogenesis Induced by Low-Sodium Diet in LDL Receptor Knockout Mice
Source: Antioxidants (Basel). 2022 Oct 13;11(10):2023. doi: 10.3390/antiox11102023 (PMC9598599; doi:10.3390/antiox11102023)
Supplement: Supplementary file 1 [file antioxidants-11-02023-s001.zip › antioxidants-1907019-supplementary.pdf]

1.1. Blood Samples Collection and Hematocrit

Biochemical determinations were performed in blood samples (200 µl) after a 12-h overnight fasting period. Blood was drawn from the caudal vein into heparinized micro-hematocrit capillary tubes. Plasma samples were obtained by blood centrifugation at 3000 rpm, at 4°C, for 20 min. Hematocrit was determined by microhematocrit method after centrifuging at 12.000 rpm, for x 30 min.

1.2. Biochemical Analyses

Plasma total cholesterol (TC) and triglyceride (TG) concentrations were determined by enzymatic colorimetric kits (Labtest, Lagoa Santa, MG, Brazil). Blood glucose concentration was assessed by Accu Check® Performa glucometer (Roche, Sao Paulo, SP, Brazil).

1.3. Lipoprotein Profile

The plasma lipoprotein profile (VLDL, LDL, HDL) was determined by fast protein liquid chromatography (FPLC) in the final period of the study. Plasma samples (100 µl) were injected into Superose HR 10/30 6 column (FPLC System, Pharmacia, Upsalla, Sweden) and eluded, in constant flow of 0.5mL/min, with Tris buffer (Tris 10 nM, NaCl 150 mM, EDTA 1mM and NaN3 0.03% - pH 7.2). The lipoprotein cholesterol and TG contents were determined by enzymatic colorimetric kits (Labtest, Lagoa Santa, MG, Brazil).

1.4. Urinary Sodium Assessment

Twenty-four-hour urinary samples were individually obtained by metabolic cages. Urinary sodium (UNa) concentration was assessed by FC 280 flame spectrophotometer (CELM; São Paulo, Brazil).

1.5. Blood Pressure Measurement

Systolic blood pressure (SBP) was measured by tail-cuff plethysmography using Visitech Systems (model BP-2000-M2 Blood Pressure Analysis System-Apex, NC, USA).

After mouse preconditioning to the measurement system, eight readings were recorded on two consecutive days and averaged to obtain the mean values.

Table S1. Antibodies used in the immunofluorescence staining.

**Target antigen**	**Vendor or Source**	**Catalog #**	**Working concentration**
Anti-4 Hydroxynonenal antibody	Abcam, Cambridge, UK	ab46545	(1:50)
Anti-Angiotensin II Type 1 Receptor antibody	Abcam, Cambridge, UK	ab18801	(1:50)
Anti-Carboxymethyl Lysine antibody	Abcam, Cambridge, UK	Ab27684	(1:30)

Anti-AGER/RAGE (RABBIT) antibody	Rockland Immunochemicals Inc, USA	600-401-P67	(1:10)

Table S2: Description of the experimental diets.

Description	Source / Repository
Low-sodium diet (LS; 0.06% sodium = 0.15% NaCl)	Envigo Teklad Diets - Indianapolis, Indiana, USA - TD 92141
Normal-sodium diet (NS; 0.5% sodium = 1.27% NaCl)	Envigo Teklad Diets - Indianapolis, Indiana, USA - TD92140
